# KLK3 in the Regulation of Angiogenesis—Tumorigenic or Not?

**DOI:** 10.3390/ijms222413545

**Published:** 2021-12-17

**Authors:** Hannu Koistinen, Jaana Künnapuu, Michael Jeltsch

**Affiliations:** 1 Department of Clinical Chemistry, Helsinki University Hospital and University of Helsinki, 00290 Helsinki, Finland; 2 Drug Research Program, University of Helsinki, 00014 Helsinki, Finland; jaana.vulli@helsinki.fi; 3 Individualized Drug Therapy Research Program, University of Helsinki, 00014 Helsinki, Finland; 4 Wihuri Research Institute, 00290 Helsinki, Finland

**Keywords:** KLK3, PSA, VEGF-C, VEGF-D, proteolysis, angiogenesis, cancer

## Abstract

In this focused review, we address the role of the kallikrein-related peptidase 3 (KLK3), also known as prostate-specific antigen (PSA), in the regulation of angiogenesis. Early studies suggest that KLK3 is able to inhibit angiogenic processes, which is most likely dependent on its proteolytic activity. However, more recent evidence suggests that KLK3 may also have an opposite role, mediated by the ability of KLK3 to activate the (lymph)angiogenic vascular endothelial growth factors VEGF-C and VEGF-D, further discussed in the review.

## 1. Introduction

In humans, about 600 different proteases have been identified, representing roughly 3% of protein-coding genes [1]. Collectively this set of proteases is called “degradome”. Proteases are involved in the control of multiple biological processes in all living organisms [2,3]. Several proteases are differentially expressed in cancer and are implicated in cancer progression, having roles in essentially all stages of tumor development [3,4,5,6]. While cancer has been thought to be primarily associated with increased proteolytic activity, opposite effects have also been observed for some proteases. Indeed, proteases may act also as tumor suppressors, e.g., by suppressing angiogenesis [7]. About half of the human proteases are expressed in the prostate [8]. Among these especially highly expressed are several members of the kallikrein-related peptidase (KLK) family of 15 serine proteases [9]. KLK family proteases have been suggested to function in proteolytic cascades and many are regulated by androgens in the prostate [9,10,11]. Several KLKs activate growth factors and protease-associated receptors (PARs), leading to a wide range of responses, which in many cases have been found to promote tumor growth and metastatic dissemination [12,13,14,15]. KLKs are able to cleave also several other prostate cancer-relevant substrates [9]. Among KLKs especially KLK3 is highly expressed in the prostate, being one of the most abundant prostatic proteins, while in other tissues the expression is much lower if present at all [16]. KLK3 is a secreted protease, more widely known as a prostate cancer marker, prostate-specific antigen (PSA) [17]. It is released in high levels into prostatic fluid, which, during ejaculation, mixes with other secretions to form the seminal fluid. In seminal fluid, KLK3 demonstrates highly efficient chymotrypsin-like proteolytic cleavage of semenogelins, which are the major gel-forming proteins in ejaculate [18,19]. This leads to the solubilization of the seminal fluid clot, a proposed physiological role of KLK3, and the release of the spermatozoa [9,18]. Apart from semenogelins, the activity of KLK3 towards identified substrate proteins is often very modest [20,21]. However, since the levels of proteolytically active KLK3 in extracellular fluid in the prostate can be as high as 2 µM [22] even a low activity may be significant.

In prostate cancer and some non-malignant conditions, such as benign prostatic hyperplasia and prostatitis, KLK3 is also released into the blood circulation [17]. In blood, most of the KLK3 is inactive, mostly complexed with protease inhibitors [23,24]. The diagnostic/prognostic roles of KLK3 (PSA) and its different complexed and free forms have been reviewed elsewhere [17,25,26,27].

Several cellular and xenograft model studies suggest that KLK3 promotes the growth of prostate cancer cells and tumors [28,29,30]. It should be noted that prostate cancer cell lines and xenograft tumors derived from such cell lines do not recapitulate well prostate cancer and its various subtypes [31]. Thus, the relevance of these results for various subtypes of prostate cancer is currently somewhat open. Gene ablation studies of *KLK3* cannot be performed in current animal models, as such animals lack the *KLK3* gene [9]. To supplement and overcome some of the limitations of the cell line studies, transgenic mice expressing active KLK3 in the prostate have been created [32]. Studies with these mice suggest that KLK3 is not able to initiate cancer [32]. However, the KLK3 levels in these mice were orders of magnitude lower than those in the human prostate and the potential role of KLK3 in cancer progression was not addressed. These and other proposed functions of KLK3 that promote or inhibit tumor growth and metastasis have been reviewed elsewhere [9,12,25,28,33,34,35].

In this review, we concentrate on the proposed roles of KLK3 in the regulation of angiogenesis, with a special focus on prostate cancer. The notion that tumors are no different from other organs in their need for oxygen and nutrients was first put forward by Judah Folkman in 1971, with the implication that blocking blood vessel growth could be a universal strategy to combat solid tumors [36]. In 2004, the first antiangiogenic cancer drug, bevacizumab, was approved by the FDA for the treatment of metastatic colorectal cancer in combination with a 5-fluorouracil-based chemotherapy [37]. The drug’s impact was less lasting and universal as initially hoped for [38], but anti-angiogenesis therapy has made advances since with the recognition that there is not only one “tumor angiogenesis factor” [39], and that antiangiogenic treatment might work differently than by starvation [40]. In fact, bispecific antibodies targeting two independent angiogenic pathways [the vascular endothelial growth factor (VEGF) receptor (VEGFR)/VEGF axis and the Tie/Ang pathways] appear to outperform agents that target single pathways both in preclinical oncologic [41] and non-oncologic [42] applications, as well as in clinical intraocular applications with the newly approved drug faricimab [43,44].

Over the last two decades, important roles of the lymphangiogenic VEGF growth factors (VEGF-C and VEGF-D) have been uncovered. In 2001, the lymphatic vasculature with its primary lymphangiogenic growth factor VEGF-C was recognized as a prerequisite for lymphatic metastasis in a variety of mouse tumor models [45,46,47]. Targeting lymphatics to prevent metastasis was, perhaps luckily, never seriously attempted, as subsequent data have shown that immune responses against tumors are crucially dependent on the lymphatic drainage of the tumor or its immediate vicinity [48]. However, some conditions such as proliferative diabetic retinopathy (PDR) or wet age-related macular degeneration (AMD) might benefit from a simultaneous blocking of VEGF-A, VEGF-C, and VEGF-D, if anti-VEGF-A therapy alone appears insufficient [49]. Indeed, a suitable biologic, the VEGF-C/VEGF-D trap OPT-302, has recently been fast-tracked by the U.S. Food and Drug Administration (FDA) for the treatment of wet AMD, and clinical trials studying combination treatments of OPT-302 with anti-VEGF-A agents are ongoing [50].

## 2. The Role of KLK3 in Angiogenesis

Antiangiogenic activity of KLK3 was first reported by Fortier and coworkers in 1999 [51]. To this end, they deployed various assays using endothelial cells. They showed that KLK3, at concentrations found in the prostate, inhibited VEGF or fibroblast growth factor 2 (FGF-2) stimulated proliferation, migration and invasion of human umbilical vein endothelial cells (HUVECs). The antiproliferative effect was also shown using bovine adrenal capillary endothelial cells (BCEC) and human dermal microvascular endothelial cells (HDMEC). Further, KLK3 demonstrated also inhibitory activity on a widely used endothelial cell tube formation assay for the angiogenic potential of endothelial cells. In this assay, endothelial cells (HUVECs) grown on top of a basement membrane preparation (e.g., Matrigel) undergo morphological differentiation into capillary (or tube)-like structures [52]. In a follow-up study, Fortier and colleagues showed that KLK3 inhibits angiogenesis also in the in vivo Matrigel plug model [53]. In that model, FGF-2 containing Matrigel was injected subcutaneously into mice, followed by subcutaneous daily injection of KLK3. About a week later, mice were euthanized and the Matrigel plugs were removed for quantitation of blood vessel growth by measuring the hemoglobin content of the plugs. Since then, several studies have confirmed the antiangiogenic activity of KLK3 in the HUVEC tube formation model with sub-physiological KLK3 concentrations [30,54,55]. KLK3 has also been found to inhibit capillary-like sprouting of HUVECs grown on beads in fibrin gels [56].

### 2.1. Mechanisms and Requirement of Proteolytic Activity

The mechanism by which KLK3 exerts its antiangiogenic effect in these models is still unclear. Studies utilizing KLK3-isoforms with different proteolytic activity (like the inactive proform and internally cleaved KLK3 [54], and a less active KLK3-isoform with an amino acid change due to a polymorphism in the *KLK3* gene [30]), KLK3-inhibitors [51,54,56,57] and -stimulators [58] all suggest that proteolytic activity of KLK3 is closely related to the antiangiogenic activity. However, some studies suggest that also a proteolytically inactive KLK3 retains antiangiogenic activity [53,59]. It has also been described that 11–17 amino acid long peptides, representing the areas (on a linear sequence) that are predicted to be on the surface of KLK3 molecule and hypothesized to be capable of interacting with unknown cell surface targets, have the antiangiogenic activity [56]. It is somewhat difficult to understand why two separate linear peptides would have such an activity. Since the purification of the peptides was not described in the paper, one possibility is that the peptide preparations contained impurities, not equally present in different preparations, interfering with cellular assays. Such impurities are not uncommon [60].

Substrate proteins that may mediate the antiangiogenic activity of KLK3 have been described. These include plasminogen, which has been reported to be converted by KLK3 into angiostatin-like fragments, capable of inhibiting endothelial cell tube formation [61]. This has been questioned and may be related to impurities, frequently observed in KLK3 preparations [21,62]. Another potential mediator cleaved by KLK3 is galectin-3, which promotes angiogenesis [21,63,64]. However, the addition of neither full length nor KLK3-derived fragments of these proteins was found to have an effect on the HUVEC tube formation model, in which KLK3 decreases tube formation [21]. To date, perhaps the most interesting substrates that link KLK3 to the regulation of angiogenesis are VEGF-C and VEGF-D [65].

### 2.2. Activation of the VEGF-C and VEGF-D

Proteolytic processing removes the C-terminal domains of both the classic hemangiogenic VEGF (VEGF-A) and the lymphangiogenic VEGF-C/D, which results in a loss of binding to extracellular matrix and cell surface proteolycans [66]. VEGF-C and VEGF-D do undergo also another cleavage, which distinguishes them from the other VEGF family members. This cleavage occurs N-terminally of their receptor binding domain, removes their unique N-terminal propeptides, and, most importantly, enables these growth factors in the first place to activate their receptors. Before this “activating” cleavage, VEGF-C is known as pro-VEGF-C. Pro-VEGF-C it is not only unable to activate its receptors, but acts as a competitive inhibitor of its mature counterpart in receptor activation [67].

Five different proteases have been reported to activate VEGF-C: plasmin, ADAMTS3, KLK3, cathepsin D (CTSD), and thrombin. ADAMTS3 is the physiological activator of VEGF-C during embryonic development [68,69], whereas thrombin and plasmin are thought to act concertedly during wound healing to re-establish vascularization [70]. Intriguingly, both KLK3 and CTSD were identified as potent proteolytic activators of VEGF-C and VEGF-D [65]. To this end purified KLK3 was used and, to further prove that KLK3 was responsible for the activating cleavage, a monoclonal antibody inhibiting KLK3 was used to abolish the effect. The cleavage took place after a tyrosine residue, agreeing with the chymotrypsin-like activity of KLK3. Furthermore, the cleavage led to the activation of these growth factors, which was shown in a series of cell-based assays [65].

**Figure 1:**
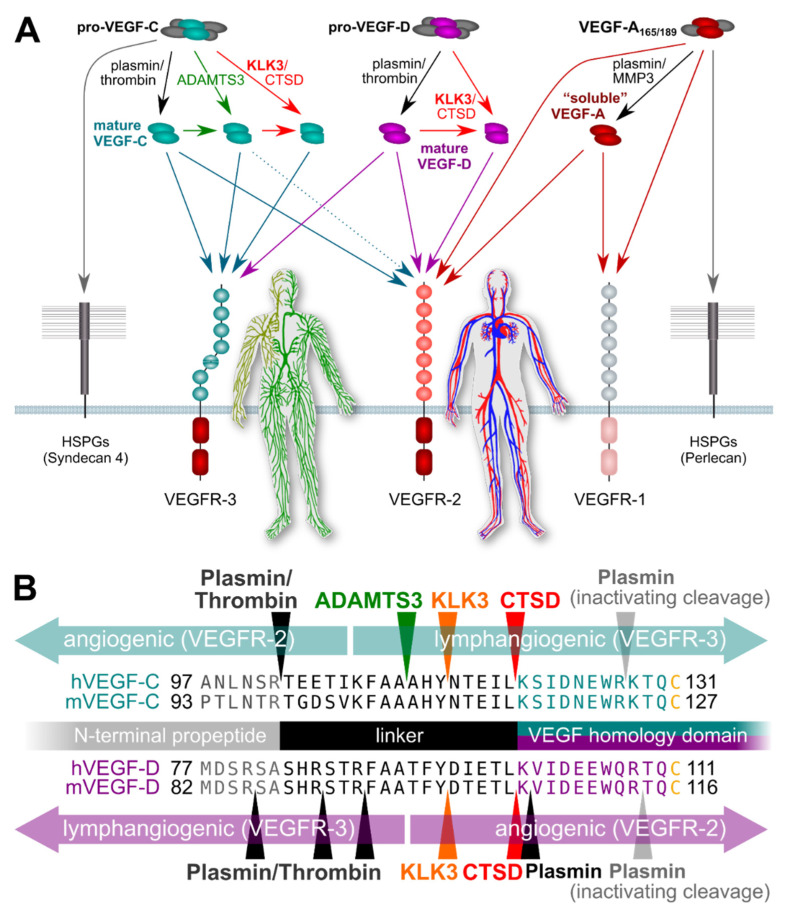
Simplified schematic of the proteolytic processing of VEGFs. (**A**) In vitro data on the properties of different forms of mature VEGF-C and VEGF-D predict that KLK3 promotes lymphangiogenesis in tumors overexpressing VEGF-C, but angiogenesis in tumors overexpressing VEGF-D. Lymphangiogenesis is mediated via VEGFR-3, and progressive proteolytic processing renders VEGF-C selective for VEGFR-3. Unlike VEGF-C, VEGF-D can become exclusively angiogenic upon proteolytic processing [75]. VEGF-C- and VEGF-D mediated lymphangiogenesis could enhance tumor immune surveillance during the early stages of tumor development but promote metastasis (VEGF-C) or tumor angiogenesis (VEGF-D) during later stages. In the absence of proteolytic processing, the longer isoforms of VEGF-A, pro-VEGF-D and pro-VEGF-C are sequestered on cell surface heparan sulfate proteolglycans (HSPGs) and in the extracellular matrix. (**B**) The locations of proteolytic processing for proteases known to cleave VEGF-C and/or VEGF-D. Note that the processing sites for plasmin and thrombin for VEGF-D have not been experimentally determined and are only predicted based on the known cleavage specificities.

Studies supporting an association of KLK3 with metastasis have mostly been performed in vitro, focusing on the proteolytic degradation of the extracellular matrix facilitating tumor cell invasion [33,71]. However, the potential of KLK3 to stimulate tumor-associated angiogenesis and lymphangiogenesis via VEGF-C and VEGF-D activation does provide another possible mechanism by which KLK3 may support metastatic dissemination of cancer cells [65]. Blood vessel and lymphatic growth are mediated by two different receptors (VEGFR-2 and VEGFR-3, respectively). Both VEGF-C and VEGF-D do activate these two receptors [66], but their affinity towards VEGFR-2 and VEGFR-3 differs depending on which enzymes catalyze their activation (Figure 1). VEGF-C and VEGF-D can activate both VEGFR-2 and VEGFR-3 when they are activated by plasmin or thrombin. However, VEGF-D loses most of its lymphangiogenic signaling capacity upon cleavage by KLK3 or CTSD [65], while maintaining most of its angiogenic signaling potential, thus, resembling tumor-derived VEGF-D [72]. VEGF-C behaves in exactly the opposite way: it loses most of its angiogenic potential when activated by KLK3 or CTSD, but retains most of its lymphangiogenic potential [65,66]. Of note, none of the currently used antibodies for VEGF-C and VEGF-D detection discriminates between the inactive pro-forms and the active, mature forms of the growth factors, or between the different mature forms (discussed more below). This fact perhaps explains most of the controversial data in clinical studies that have aimed to establish links between VEGF-C levels and prostate cancer progression [73,74]. In addition, VEGF-C and VEGF-D expression might very well differ between individual cancers and during cancer progression, questioning the predictive value of any single marker to address different subtypes of prostate cancer, which often is also multifocal. Thus, more detailed studies on this are called for.

A recent analysis of interactions between *KLK3* single nucleotide polymorphisms (SNPs) and other SNPs to identify interactions associated with prostate cancer aggressiveness support the notion that KLK3 and VEGF-C concertedly are involved in the regulation of prostate cancer tumor angiogenesis. Although not common, the *VEGFC/KLK3* SNP–SNP interaction pair rs174776/rs3775202 was highly significantly associated with prostate cancer aggressiveness [76]. Since both SNPs are intronic and not predicted to change mRNA splicing, their effect is likely regulatory. The same *KLK3* SNP is also associated with a decrease in male fertility [77].

### 2.3. Clinical Observations Relating to the Role of KLK3 in the Regulation of Angiogenesis

Prostate cancer tumors typically grow very slowly after reaching the size which requires a blood supply to support the tumor growth. Providing that KLK3 has antiangiogenic activity in prostate cancer, the slow growth of prostate cancer could be related to the antiangiogenic activity of KLK3 [25]. Indeed, in prostate cancer tumors, high KLK3 expression has been found to be associated with low angiogenesis activity (as determined by CD34 staining) [78] and microvessel density (determined by CD31 staining) [79]. KLK3 expression is often reduced upon loss of differentiation in cancer cells and low KLK3 levels in the prostate are associated with poor prognosis [80,81,82]. The serum concentrations of KLK3 do not correlate with the expression in the prostate as the increased serum levels in cancer are due to an increased release of KLK3 into blood [17]. Still, it is noteworthy that KLK3 levels in serum are sometimes elevated even decades before the development of otherwise detectable tumors [24,83]. Thus, the clinical studies may be interpreted to support a dual role for KLK3 in prostate cancer, i.e., KLK3 may favor tumor development at early stages of prostate cancer and later inhibit tumor growth by its antiangiogenic activity [25]. However, this remains to be confirmed. If the effect of KLK3 on tumor progression is dependent on its ability to activate VEGF-C and VEGF-D, the net KLK3 effect would depend on the tumor’s expression levels of VEGF-C, VEGF-D and other proteases that are able to regulate their activity. Notably, high levels of plasmin can completely inactivate both VEGF-C and VEGF-D [67].

### 2.4. Regulation of VEGFs and KLK3 in Relation to Prostate Cancer Progression

Not surprisingly, VEGF-A and VEGF-C are differently regulated in response to hypoxia, inflammatory signals and sex hormones. Especially the hormones, and more specifically androgens, such as testosterone, have a central role in prostate cancer and also in the regulation of KLK3 expression [9,84]. Prostate cancers are initially responsive to androgen ablation, but typically such cancers progress to so-called castration-resistant prostate cancer (CRPC) due to restored androgen receptor signaling via various mechanisms [84,85]. Such prostate cancers are treatment-resistant and metastasis-prone. Typically, also KLK3 expression and blood levels, which were initially down-regulated by androgen-ablation, start to rise again upon the development of CRPC [17]. Moreover, like solid tumors in general, prostatic tumors are hypoxic [86] and associated with inflammation [87]. Thus, it is relevant to consider the regulation of VEGFs in the context of prostate cancer to further understand the role of KLK3 activation of VEGF-C and -D.

The blood endothelium-specific VEGF-A is mostly regulated at the mRNA level [88]. When tissue oxygen supply is insufficient, VEGF-A production is ramped up, leading, under normal conditions, to vascular growth and normalization of oxygen supply. VEGF-C production, on the other hand, is regulated by proinflammatory signals [89,90,91], and VEGF-C improves tissue clearance and immune cell trafficking via stimulation of lymphatic growth and innervation [92], limiting inflammatory responses through increased drainage [93,94] and immunomodulation [95].

While VEGF-A production is almost inevitably switched on sooner or later in the hypoxic areas of a tumor (“angiogenic switch”), it is less clear how tumors become lymphangiogenic. During the developmental expansion of the lymphatic system, increased interstitial tissue pressure dictates lymphatic vessel growth via pressure-dependent signal transduction (“mechanotransduction”) of VEGFR-3, mediated by β1-integrin and integrin-linked kinase (ILK) [96]. Tumors feature similarly increased interstitial pressure, but such pressure would act on tumor cells, and would not contribute to tumor lymphangiogenesis, as interstitial pressure has not (yet?) been seen to modulate VEGF-C expression. Interestingly, tumor cells do sometimes express VEGFR-3, including prostate cancer tumor cell lines, such as PC-3 [97], from which VEGF-C had been originally cloned [98].

The expression of VEGFs in the healthy prostate and in prostate cancer tissue has been analyzed and reviewed with a focus on VEGF-A [99,100]. VEGF-A levels in serum are increased both in prostate cancer and benign prostatic hyperplasia patients, as compared to healthy individuals, but as individual serum levels have a considerable variance, VEGF-A is not a good predictor for disease progression itself [101].

The expression of both VEGF-A and VEGF-C is hormone-sensitive. Therefore, it is affected by hormone treatment of prostate cancer. For example, VEGF-A, like KLK3, is downregulated by castration and upregulated by testosterone [17,102]. In fact, the hormone treatment response is partly mediated via the endothelium, which undergoes first regression, but recovers by a VEGF-A-mediated mechanism [103,104]. While this effect is likely indirect as endothelial cells normally do not express androgen receptors, it argues for an antiangiogenic treatment opportunity. Opposite to their long-known role in prostate cancer [85], the sex hormones have only recently received increased attention for the treatment of other diseases such as lymphedema, which can result from disturbances in the VEGF-C/VEGFR-3 signaling pathway [105].

Androgen deprivation therapy does seem to induce lymphangiogenesis in prostate cancer and, thus, could contribute to lymphatic metastasis [106] as well as to an increased immune response [48]. It is unclear how this effect is mediated and whether it is relevant for the development of aggressive CRPC. In experimental prostate tumors, tumor cells themselves have been shown to be the source of lymphangiogenic growth factors [107], but in clinical diseases, including ocular neovascularization and cervical cancer, infiltrating macrophages appear to be a major source of VEGF-C and VEGF-D [108,109]. This may as well be the case for prostate cancer [110]. In mice, VEGF-C levels decrease and VEGF-D levels increase after castration [104], rendering the net effect of angiogenesis versus lymphangiogenesis dependent on the proteases that are available for activation. Vice versa, stimulation by estradiol results in the upregulation of key lymphatic signaling molecules such as VEGF-D, VEGFR-3, and Lyve-1, while blocking estrogen receptor alpha (ER⍺) by tamoxifen leads to lymphatic degeneration [111].

Additionally, in humans, androgen deprivation therapy of prostate cancer has been shown to reduce the levels of VEGF-C and its receptor VEGFR-3 [112], but the individual net effect and the vascular response might be different for early versus late stages of the disease [113], and increased VEGF-C levels appear to dominate during the rebound and disease progression phase both in clinical studies as well as in cell culture experiments [114,115,116,117]. Similar results have also been seen in other cancer types, e.g., in breast cancer, where, despite early contradicting data, the association of high VEGF-C levels with poor survival appears to solidify with accumulating higher-quality studies [118].

**Figure 2:**
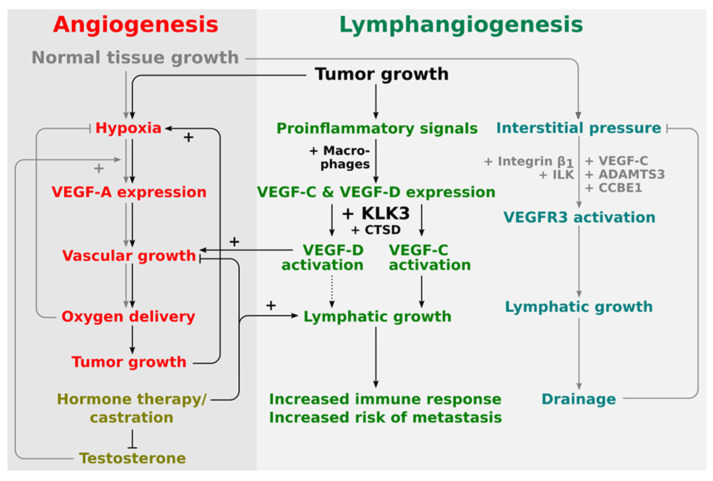
Proposed placement of KLK3 into the regulatory network of angiogenesis and lymphangiogenesis. The central stimulus for both physiological and pathological angiogenesis is hypoxia. Therefore, tumor and physiological angiogenesis share common regulatory pathways. Lymphangiogenesis in physiological and pathological settings appears to be quite differently regulated. During development, lymphangiogenesis is stimulated by the interstitial pressure which amplifies VEGFR-3 signaling via a β1-integrin/ILK-assisted mechanism. In the tumor setting, on the other hand, tumor-infiltrating macrophages are major producers of both VEGF-C and VEGF-D. Activation of VEGF-D by KLK3 and CTSD does generate a predominantly angiogenic mature VEGF-D, while activation of VEGF-C does generate a predominantly lymphangiogenic mature VEGF-C. VEGF-D can therefore replace VEGF-A (rendering anti-VEGF-A treatments ineffective), while VEGF-C leads to the ambivalent outcome of increased immune response and increased likelihood of metastasis.

Thus, it appears that KLK3 and VEGF-C, which KLK3 is able to activate, are, at least partially, similarly regulated by androgens in prostate cancer. This supports the potential role of KLK3 as a VEGF-C activator in prostate cancer and that they may act in a concerted fashion to drive prostate cancer progression. Less is known about the role of VEGF-D in this respect, but based on the in vitro data, KLK3 would likely mediate its angiogenic effects via VEGF-D, while its lymphangiogenic effects would be mediated via VEGF-C (see Figure 2).

### 2.5. Detecting VEGFs and Measuring VEGF Levels

Studies that aim to associate VEGF levels with clinical outcomes are common and important for understanding the functional role(s) of VEGFs in prostate cancer. However, those are not without pitfalls, which we briefly address here, given the importance of the topic. Although relatively easy to perform, mRNA quantification is just a proxy for the actual growth factor concentration, the reliable measurement of the protein levels itself being more difficult, and even more so for the active form of the growth factors. Naturally the same applies to proteases, including KLK3, which are often expressed as proenzymes or zymogens and activated by other proteases. Antibody-based detection (ELISA, Western blot, immunohistochemistry) stands and falls with the quality of the antibody. In a small study of commercially available VEGF-C antibodies, roughly half of the antibodies completely failed to detect a mixture of inactive pro-VEGF-C and activated VEGF-C [65].

In addition, most VEGFs exist in multiple different isoforms created either by differential mRNA splicing or by proteolytic processing [66], and even high-quality validated antibodies from reputable vendors are validated usually only for the most common, canonical isoform.

On top of the above limitations, we need to acknowledge the fact that VEGF-C and VEGF-D are produced as precursors, which are unable to activate their corresponding receptors. Measuring the ratio of inactive to active growth factor would account for this if precursor-specific antibodies were available. The pro-forms of VEGF-C and VEGF-D do not appear in large quantities in the soluble phase but are rather deposited to cell surfaces or the extracellular matrix [119]. Whether this provides a way to detect activated forms is still a rather theoretical argument. Noteworthy, since all VEGFs are secreted, their retention in histological sections or in immunofluorescence is difficult to control for. Hence, stainings often focus on intracellular or extracellular matrix staining, but their relevance is not well known. Moreover, there are no data about what forms of these growth factors can be found in blood plasma or serum and how the forms measured by the current assays relate to the bioavailability of these growth factors in vivo.

## 3. Concluding Remarks

Most of the early studies evaluating the antiangiogenic activity of KLK3 have been carried out with widely used tube formation assays using HUVECs. However, the model has several limitations, especially in the context of cancer, e.g., the model is not able to address fully the angiogenic process and HUVECs are very different from the endothelial cells in cancer. Therefore, it is not surprising that, using other models, apparently opposite effects for KLK3 have also been proposed, i.e., the promotion of tumor angiogenesis via the activation of VEGF-C and VEGF-D. The importance of VEGF-C and VEGF-D for tumor lymphangiogenesis has been demonstrated 20 years ago [45,120], but at the time, none of the VEGF-C- or VEGF-D-activating proteases had been identified. Proteolytic processing is a prerequisite for the effects of VEGF-C and VEGF-D [121]. The tumor environment does provide for a rich and heterogeneous source of different proteases. Which one(s) of the VEGF-C/-D-activating proteases (including several that have been identified, but not yet reported) will be confirmed as relevant for tumor (lymph)angiogenesis is still unclear. However, such a protease would be interesting not only as a prognostic marker, but also as a drug target. Recent data suggest that, at least in certain tumor types, the angiogenic form of VEGF-D, identical or similar to the KLK3 activated VEGF-D, is responsible for the tumor becoming refractory to anti-VEGF-A treatment [122], and activated VEGF-C may rather act as a lymphangiogenic factor than an angiogenic one [72]. Thus, the antiangiogenic activity of KLK3, observed in the tube formation model and other early studies, and the more recently found activation of VEGF-C and -D by KLK3 are not necessarily conflicting.

Finally, it should be noted that in addition to the traditional neovascularization by sprouting angiogenesis, tumors may utilize several different “non-orthodox” mechanisms to establish the blood circulation needed for growth. These include intussusception (angiogenesis by vessel splitting), vessel co-option, recruitment of bone-marrow-derived endothelial progenitors, and vascular mimicry, e.g., by (transdifferentiated) tumor cells [39]. Thus, single cellular models are likely not able to predict the consequences at the tumor or organism level. Furthermore, as the transcription of androgen receptor-regulated genes is greatly dependent on the environment, single-cell models also fail to recapitulate the androgen responsiveness observed in vivo [123]. While the animal models should be valuable, those may lack some of the relevant proteins, such as KLK3 itself, which is not expressed in most of the widely used animal models, and its substrates. We foresee that the modern three-dimensional tumor angiogenesis models would be useful for the elucidation of the role of KLK3 in tumor angiogenesis. Such models incorporate, with cancer cells, tumor microenvironment, including extracellular matrix, and endothelial and other stromal cells, or their progenitor cells [124,125,126,127]. However, for the most part, such models remain to be established for studies of prostate cancer angiogenesis. We also advocate the use of several different models for studying such a complex phenomenon as tumor angiogenesis. Still, the studies indicating a role of KLK3 in the regulation of angiogenesis, together with the clinical observations and similar regulation of KLK3 and VEGF-C by androgens, make a strong case for KLK3 having a complex role in the regulation of (lymph)angiogenesis. The consequence of such regulation, together with other proposed activities of KLK3, in prostate cancer is yet to be fully determined.

## Data Availability

Not applicable.

## References

[1] Pérez-Silva J.G., Español Y., Velasco G., Quesada V. (2016). The Degradome Database: Expanding Roles of Mammalian Proteases in Life and Disease. Nucleic Acids Res..

[2] Bond J.S. (2019). Proteases: History, Discovery, and Roles in Health and Disease. J. Biol. Chem..

[3] Lopez-Otin C., Bond J.S. (2008). Proteases: Multifunctional Enzymes in Life and Disease. J. Biol. Chem..

[4] Dudani J.S., Warren A.D., Bhatia S.N. (2018). Harnessing Protease Activity to Improve Cancer Care. Annu Rev. Cancer Biol..

[5] Sevenich L., Joyce J.A. (2014). Pericellular Proteolysis in Cancer. Genes Dev..

[6] Affara N.I., Andreu P., Coussens L.M. (2009). Delineating Protease Functions during Cancer Development. Methods Mol. Biol..

[7] Lopez-Otin C., Matrisian L.M. (2007). Emerging Roles of Proteases in Tumour Suppression. Nat. Rev. Cancer.

[8] Kappelhoff R., Puente X.S., Wilson C.H., Seth A., López-Otín C., Overall C.M. (2017). Overview of Transcriptomic Analysis of All Human Proteases, Non-Proteolytic Homologs and Inhibitors: Organ, Tissue and Ovarian Cancer Cell Line Expression Profiling of the Human Protease Degradome by the CLIP-CHIP^TM^ DNA Microarray. Biochim. Biophys. Acta Mol. Cell Res..

[9] Lawrence M.G., Lai J., Clements J.A. (2010). Kallikreins on Steroids: Structure, Function, and Hormonal Regulation of Prostate-Specific Antigen and the Extended Kallikrein Locus. Endocr Rev..

[10] Lovell S., Zhang L., Kryza T., Neodo A., Bock N., De Vita E., Williams E.D., Engelsberger E., Xu C., Bakker A.T. (2021). A Suite of Activity-Based Probes to Dissect the KLK Activome in Drug-Resistant Prostate Cancer. J. Am. Chem. Soc..

[11] Pampalakis G., Sotiropoulou G. (2007). Tissue Kallikrein Proteolytic Cascade Pathways in Normal Physiology and Cancer. Biochim. Biophys. Acta.

[12] Filippou P.S., Karagiannis G.S., Musrap N., Diamandis E.P. (2016). Kallikrein-Related Peptidases (KLKs) and the Hallmarks of Cancer. Crit. Rev. Clin. Lab. Sci..

[13] Eftekhari R., de Lima S.G., Liu Y., Mihara K., Saifeddine M., Noorbakhsh F., Scarisbrick I.A., Hollenberg M.D. (2018). Microenvironment Proteinases, Proteinase-Activated Receptor Regulation, Cancer and Inflammation. Biol. Chem..

[14] Oikonomopoulou K., Diamandis E.P., Hollenberg M.D. (2010). Kallikrein-Related Peptidases: Proteolysis and Signaling in Cancer, the New Frontier. Biol. Chem..

[15] Ramachandran R., Altier C., Oikonomopoulou K., Hollenberg M.D. (2016). Proteinases, Their Extracellular Targets, and Inflammatory Signaling. Pharmacol. Rev..

[16] Shaw J.L., Diamandis E.P. (2007). Distribution of 15 Human Kallikreins in Tissues and Biological Fluids. Clin. Chem..

[17] Lilja H., Ulmert D., Vickers A.J. (2008). Prostate-Specific Antigen and Prostate Cancer: Prediction, Detection and Monitoring. Nat. Rev. Cancer.

[18] Lilja H. (1985). A Kallikrein-like Serine Protease in Prostatic Fluid Cleaves the Predominant Seminal Vesicle Protein. J. Clin. Investig..

[19] Lilja H., Abrahamsson P.A., Lundwall A. (1989). Semenogelin, the Predominant Protein in Human Semen. Primary Structure and Identification of Closely Related Proteins in the Male Accessory Sex Glands and on the Spermatozoa. J. Biol. Chem..

[20] Robert M., Gibbs B.F., Jacobson E., Gagnon C. (1997). Characterization of Prostate-Specific Antigen Proteolytic Activity on Its Major Physiological Substrate, the Sperm Motility Inhibitor Precursor/Semenogelin I. Biochemistry.

[21] Mattsson J.M., Ravela S., Hekim C., Jonsson M., Malm J., Närvänen A., Stenman U.-H., Koistinen H. (2014). Proteolytic Activity of Prostate-Specific Antigen (PSA) towards Protein Substrates and Effect of Peptides Stimulating PSA Activity. PLoS ONE.

[22] Denmeade S.R., Sokoll L.J., Chan D.W., Khan S.R., Isaacs J.T. (2001). Concentration of Enzymatically Active Prostate-Specific Antigen (PSA) in the Extracellular Fluid of Primary Human Prostate Cancers and Human Prostate Cancer Xenograft Models. Prostate.

[23] Lilja H. (2008). Testing New PSA Subforms to Enhance the Accuracy of Predicting Cancer Risk and Disease Outcome in Prostate Cancer. Clin. Chem..

[24] Stenman U.H., Hakama M., Knekt P., Aromaa A., Teppo L., Leinonen J. (1994). Serum Concentrations of Prostate Specific Antigen and Its Complex with Alpha 1-Antichymotrypsin before Diagnosis of Prostate Cancer. Lancet.

[25] Koistinen H., Stenman U.-H., Magdolen V., Sommerhoff C., Fritz H., Schmitt M. (2012). PSA (Prostate-Specific Antigen) and other Kallikrein-related Peptidases in Prostate Cancer. Kallikrein-Related Peptidases: Novel Cancer-Related Biomarkers.

[26] Loeb S., Lilja H., Vickers A. (2016). Beyond Prostate-Specific Antigen: Utilizing Novel Strategies to Screen Men for Prostate Cancer. Curr. Opin. Urol..

[27] Van Poppel H., Roobol M.J., Chapple C.R., Catto J.W.F., N’Dow J., Sønksen J., Stenzl A., Wirth M. (2021). Prostate-Specific Antigen Testing as Part of a Risk-Adapted Early Detection Strategy for Prostate Cancer: European Association of Urology Position and Recommendations for 2021. Eur. Urol..

[28] Williams S.A., Jelinek C.A., Litvinov I., Cotter R.J., Isaacs J.T., Denmeade S.R. (2011). Enzymatically Active Prostate-Specific Antigen Promotes Growth of Human Prostate Cancers. Prostate.

[29] Niu Y., Yeh S., Miyamoto H., Li G., Altuwaijri S., Yuan J., Han R., Ma T., Kuo H.C., Chang C. (2008). Tissue Prostate-Specific Antigen Facilitates Refractory Prostate Tumor Progression via Enhancing ARA70-Regulated Androgen Receptor Transactivation. Cancer Res..

[30] Srinivasan S., Stephens C., Wilson E., Panchadsaram J., DeVoss K., Koistinen H., Stenman U.-H., Brook M.N., Buckle A.M., Klein R.J. (2019). Prostate Cancer Risk-Associated Single-Nucleotide Polymorphism Affects Prostate-Specific Antigen Glycosylation and Its Function. Clin. Chem..

[31] Risbridger G.P., Toivanen R., Taylor R.A. (2018). Preclinical Models of Prostate Cancer: Patient-Derived Xenografts, Organoids, and Other Explant Models. Cold Spring Harb. Perspect. Med..

[32] Williams S.A., Xu Y., Marzo A.M.D., Isaacs J.T., Denmeade S.R. (2010). Prostate-Specific Antigen (PSA) Is Activated by KLK2 in Prostate Cancer Ex Vivo Models and in Prostate-Targeted PSA/KLK2 Double Transgenic Mice. Prostate.

[33] LeBeau A.M., Kostova M., Craik C.S., Denmeade S.R. (2010). Prostate-Specific Antigen: An Overlooked Candidate for the Targeted Treatment and Selective Imaging of Prostate Cancer. Biol. Chem..

[34] Avgeris M., Scorilas A. (2016). Kallikrein-Related Peptidases (KLKs) as Emerging Therapeutic Targets: Focus on Prostate Cancer and Skin Pathologies. Expert Opin. Ther. Targets.

[35] Moradi A., Srinivasan S., Clements J., Batra J. (2019). Beyond the Biomarker Role: Prostate-Specific Antigen (PSA) in the Prostate Cancer Microenvironment. Cancer Metastasis Rev..

[36] Folkman J. (1971). Tumor Angiogenesis: Therapeutic Implications. N. Engl. J. Med..

[37] Ellis L.M., Kirkpatrick P. (2005). Bevacizumab. Nat. Rev. Drug Discov..

[38] Moserle L., Jiménez-Valerio G., Casanovas O. (2014). Antiangiogenic Therapies: Going beyond Their Limits. Cancer Discov..

[39] Lugano R., Ramachandran M., Dimberg A. (2020). Tumor Angiogenesis: Causes, Consequences, Challenges and Opportunities. Cell. Mol. Life Sci..

[40] Goel S., Duda D.G., Xu L., Munn L.L., Boucher Y., Fukumura D., Jain R.K. (2011). Normalization of the vasculature for treatment of cancer and other diseases. Physiol. Rev..

[41] Mueller T., Freystein J., Lucas H., Schmoll H.-J. (2019). Efficacy of a Bispecific Antibody Co-Targeting VEGFA and Ang-2 in Combination with Chemotherapy in a Chemoresistant Colorectal Carcinoma Xenograft Model. Molecules.

[42] Lundh von Leithner P., Iwata D., Ng Y., Regula J., Hartmann G., Shima D.T. (2014). Bispecific Anti-VEGF/ANG2 Antibody Exhibits Superior Efficacy to VEGF Monotherapy in a Model of Spontaneous CNV. Investig. Ophthalmol. Vis. Sci..

[43] Nicolò M., Ferro Desideri L., Vagge A., Traverso C.E. (2021). Faricimab: An Investigational Agent Targeting the Tie-2/Angiopoietin Pathway and VEGF-A for the Treatment of Retinal Diseases. Expert Opin. Investig. Drugs.

[44] Khanani A.M., Heier J., Quezada Ruiz C., Lin H., Silverman D., Brittain C., Ives J., Swaminathan B., Basu K., Wong T.Y. (2021). Faricimab in Neovascular Age-Related Macular Degeneration: 1-Year Efficacy, Safety, and Durability in the Phase 3 TENAYA and LUCERNE Trials. Investig. Ophthalmol. Vis. Sci..

[45] Mandriota S.J., Jussila L., Jeltsch M., Compagni A., Baetens D., Prevo R., Banerji S., Huarte J., Montesano R., Jackson D.G. (2001). Vascular Endothelial Growth Factor-C-Mediated Lymphangiogenesis Promotes Tumour Metastasis. EMBO J..

[46] Karpanen T., Egeblad M., Karkkainen M.J., Kubo H., Ylä-Herttuala S., Jäättelä M., Alitalo K. (2001). Vascular Endothelial Growth Factor C Promotes Tumor Lymphangiogenesis and Intralymphatic Tumor Growth. Cancer Res..

[47] Skobe M., Hawighorst T., Jackson D.G., Prevo R., Janes L., Velasco P., Riccardi L., Alitalo K., Claffey K., Detmar M. (2001). Induction of Tumor Lymphangiogenesis by VEGF-C Promotes Breast Cancer Metastasis. Nat. Med..

[48] Fankhauser M., Broggi M.A.S., Potin L., Bordry N., Jeanbart L., Lund A.W., Costa E.D., Hauert S., Rincon-Restrepo M., Tremblay C. (2017). Tumor Lymphangiogenesis Promotes T Cell Infiltration and Potentiates Immunotherapy in Melanoma. Sci. Transl. Med..

[49] Gucciardo E., Lehti T.A., Korhonen A., Salvén P., Lehti K., Jeltsch M., Loukovaara S. (2020). Lymphatics and the eye. Duodecim.

[50] Arepalli S., Kaiser P.K. (2021). Pipeline Therapies for Neovascular Age Related Macular Degeneration. Int. J. Retin. Vitr..

[51] Fortier A.H., Nelson B.J., Grella D.K., Holaday J.W. (1999). Antiangiogenic Activity of Prostate-Specific Antigen. J. Natl. Cancer Inst..

[52] Kubota Y., Kleinman H.K., Martin G.R., Lawley T.J. (1988). Role of Laminin and Basement Membrane in the Morphological Differentiation of Human Endothelial Cells into Capillary-like Structures. J. Cell Biol..

[53] Fortier A.H., Holaday J.W., Liang H., Dey C., Grella D.K., Holland-Linn J., Vu H., Plum S.M., Nelson B.J. (2003). Recombinant Prostate Specific Antigen Inhibits Angiogenesis in Vitro and in Vivo. Prostate.

[54] Mattsson J.M., Valmu L., Laakkonen P., Stenman U.-H., Koistinen H. (2008). Structural Characterization and Anti-Angiogenic Properties of Prostate-Specific Antigen Isoforms in Seminal Fluid. Prostate.

[55] Mattsson J.M., Laakkonen P., Stenman U., Koistinen H. (2009). Antiangiogenic Properties of Prostate-----specific Antigen (PSA). Scand. J. Clin. Lab. Investig..

[56] Chadha K.C., Nair B., Godoy A., Rajnarayanan R., Nabi E., Zhou R., Patel N.R., Aalinkeel R., Schwartz S.A., Smith G.J. (2015). Anti-Angiogenic Activity of PSA-Derived Peptides. Prostate.

[57] Koistinen H., Wohlfahrt G., Mattsson J.M., Wu P., Lahdenperä J., Stenman U. (2008). Novel Small Molecule Inhibitors for Prostate-----specific Antigen. Prostate.

[58] Mattsson J.M., Närvänen A., Stenman U.-H., Koistinen H. (2012). Peptides Binding to Prostate-Specific Antigen Enhance Its Antiangiogenic Activity. Prostate.

[59] Chadha K.C., Nair B.B., Chakravarthi S., Zhou R., Godoy A., Mohler J.L., Aalinkeel R., Schwartz S.A., Smith G.J. (2011). Enzymatic Activity of Free-Prostate-Specific Antigen (f-PSA) Is Not Required for Some of Its Physiological Activities. Prostate.

[60] De Spiegeleer B., Vergote V., Pezeshki A., Peremans K., Burvenich C. (2008). Impurity Profiling Quality Control Testing of Synthetic Peptides Using Liquid Chromatography-Photodiode Array-Fluorescence and Liquid Chromatography-Electrospray Ionization-Mass Spectrometry: The Obestatin Case. Anal. Biochem..

[61] Heidtmann H.H., Nettelbeck D.M., Mingels A., Jager R., Welker H.G., Kontermann R.E. (1999). Generation of Angiostatin-like Fragments from Plasminogen by Prostate-Specific Antigen. Br. J. Cancer.

[62] Manning M.L., Kostova M., Williams S.A., Denmeade S.R. (2012). Trypsin-like Proteolytic Contamination of Commercially Available Psa Purified from Human Seminal Fluid. Prostate.

[63] Nangia-Makker P., Honjo Y., Sarvis R., Akahani S., Hogan V., Pienta K.J., Raz A. (2000). Galectin-3 Induces Endothelial Cell Morphogenesis and Angiogenesis. Am. J. Pathol..

[64] Markowska A.I., Liu F.T., Panjwani N. (2010). Galectin-3 Is an Important Mediator of VEGF- and BFGF-Mediated Angiogenic Response. J. Exp. Med..

[65] Jha S.K., Rauniyar K., Chronowska E., Mattonet K., Maina E.W., Koistinen H., Stenman U.-H., Alitalo K., Jeltsch M. (2019). KLK3/PSA and Cathepsin D Activate VEGF-C and VEGF-D. Elife.

[66] Künnapuu J., Bokharaie H., Jeltsch M. (2021). Proteolytic Cleavages in the VEGF Family: Generating Diversity among Angiogenic VEGFs, Essential for the Activation of Lymphangiogenic VEGFs. Biology.

[67] Jeltsch M., Jha S.K., Tvorogov D., Anisimov A., Leppänen V.-M., Holopainen T., Kivelä R., Ortega S., Kärpanen T., Alitalo K. (2014). CCBE1 Enhances Lymphangiogenesis via A Disintegrin and Metalloprotease with Thrombospondin Motifs-3–Mediated Vascular Endothelial Growth Factor-C Activation. Circulation.

[68] Bui H.M., Enis D., Robciuc M.R., Nurmi H.J., Cohen J., Chen M., Yang Y., Dhillon V., Johnson K., Zhang H. (2016). Proteolytic Activation Defines Distinct Lymphangiogenic Mechanisms for VEGFC and VEGFD. J. Clin. Investig..

[69] Janssen L., Dupont L., Bekhouche M., Noel A., Leduc C., Voz M., Peers B., Cataldo D., Apte S.S., Dubail J. (2016). ADAMTS3 Activity Is Mandatory for Embryonic Lymphangiogenesis and Regulates Placental Angiogenesis. Angiogenesis.

[70] Lim L., Bui H., Farrelly O., Yang J., Li L., Enis D., Ma W., Chen M., Oliver G., Welsh J.D. (2019). Hemostasis Stimulates Lymphangiogenesis through Release and Activation of VEGFC. Blood.

[71] Ishii K., Otsuka T., Iguchi K., Usui S., Yamamoto H., Sugimura Y., Yoshikawa K., Hayward S.W., Hirano K. (2004). Evidence That the Prostate-Specific Antigen (PSA)/Zn2+ Axis May Play a Role in Human Prostate Cancer Cell Invasion. Cancer Lett..

[72] Zhao Y.-C., Ni X.-J., Wang M.-H., Zha X.-M., Zhao Y., Wang S. (2012). Tumor-Derived VEGF-C, but Not VEGF-D, Promotes Sentinel Lymph Node Lymphangiogenesis Prior to Metastasis in Breast Cancer Patients. Med. Oncol..

[73] Yang Z.-S., Xu Y.-F., Huang F.-F., Ding G.-F. (2014). Associations of Nm23H1, VEGF-C, and VEGF-3 Receptor in Human Prostate Cancer. Molecules.

[74] Mori R., Dorff T.B., Xiong S., Tarabolous C.J., Ye W., Groshen S., Danenberg K.D., Danenberg P.V., Pinski J.K. (2010). The Relationship between Proangiogenic Gene Expression Levels in Prostate Cancer and Their Prognostic Value for Clinical Outcomes. Prostate.

[75] Leppanen V.-M., Jeltsch M., Anisimov A., Tvorogov D., Aho K., Kalkkinen N., Toivanen P., Ylä-Herttuala S., Ballmer-Hofer K., Alitalo K. (2011). Structural Determinants of Vascular Endothelial Growth Factor-D Receptor Binding and Specificity. Blood.

[76] Lin H.-Y., Huang P.-Y., Cheng C.-H., Tung H.-Y., Fang Z., Berglund A.E., Chen A., French-Kwawu J., Harris D., Pow-Sang J. (2021). KLK3 SNP–SNP Interactions for Prediction of Prostate Cancer Aggressiveness. Sci. Rep..

[77] Gupta N., Sudhakar D.V.S., Gangwar P.K., Sankhwar S.N., Gupta N.J., Chakraborty B., Thangaraj K., Gupta G., Rajender S. (2017). Mutations in the Prostate Specific Antigen (PSA/KLK3) Correlate with Male Infertility. Sci. Rep..

[78] Jemaa A.B., Bouraoui Y., Sallami S., Banasr A., Rais N.B., Ouertani L., Nouira Y., Horchani A., Oueslati R. (2010). Co-Expression and Impact of Prostate Specific Membrane Antigen and Prostate Specific Antigen in Prostatic Pathologies. J. Exp. Clin. Cancer Res..

[79] Papadopoulos I., Sivridis E., Giatromanolaki A., Koukourakis M.I. (2001). Tumor Angiogenesis Is Associated with MUC1 Overexpression and Loss of Prostate-Specific Antigen Expression in Prostate Cancer. Clin. Cancer Res..

[80] Abrahamsson P.A., Lilja H., Falkmer S., Wadstrom L.B. (1988). Immunohistochemical Distribution of the Three Predominant Secretory Proteins in the Parenchyma of Hyperplastic and Neoplastic Prostate Glands. Prostate.

[81] Stege R., Grande M., Carlstrom K., Tribukait B., Pousette A. (2000). Prognostic Significance of Tissue Prostate-Specific Antigen in Endocrine-Treated Prostate Carcinomas. Clin. Cancer Res..

[82] Bonk S., Kluth M., Hube-Magg C., Polonski A., Soekeland G., Makropidi-Fraune G., Möller-Koop C., Witt M., Luebke A.M., Hinsch A. (2019). Prognostic and Diagnostic Role of PSA Immunohistochemistry: A Tissue Microarray Study on 21,000 Normal and Cancerous Tissues. Oncotarget.

[83] Lilja H., Cronin A.M., Dahlin A., Manjer J., Nilsson P.M., Eastham J.A., Bjartell A.S., Scardino P.T., Ulmert D., Vickers A.J. (2011). Prediction of Significant Prostate Cancer Diagnosed 20 to 30 Years Later with a Single Measure of Prostate-Specific Antigen at or before Age 50. Cancer.

[84] Dai C., Heemers H., Sharifi N. (2017). Androgen Signaling in Prostate Cancer. Cold Spring Harb. Perspect. Med..

[85] Auchus R.J., Sharifi N. (2020). Sex Hormones and Prostate Cancer. Annu. Rev. Med..

[86] Fraga A., Ribeiro R., Príncipe P., Lopes C., Medeiros R. (2015). Hypoxia and Prostate Cancer Aggressiveness: A Tale with Many Endings. Clin. Genitourin. Cancer.

[87] De Marzo A.M., Platz E.A., Sutcliffe S., Xu J., Grönberg H., Drake C.G., Nakai Y., Isaacs W.B., Nelson W.G. (2007). Inflammation in Prostate Carcinogenesis. Nat. Rev. Cancer.

[88] Arcondéguy T., Lacazette E., Millevoi S., Prats H., Touriol C. (2013). VEGF-A MRNA Processing, Stability and Translation: A Paradigm for Intricate Regulation of Gene Expression at the Post-Transcriptional Level. Nucleic Acids Res..

[89] Baluk P., Tammela T., Ator E., Lyubynska N., Achen M.G., Hicklin D.J., Jeltsch M., Petrova T.V., Pytowski B., Stacker S.A. (2005). Pathogenesis of Persistent Lymphatic Vessel Hyperplasia in Chronic Airway Inflammation. J. Clin. Investig..

[90] Krebs R., Tikkanen J.M., Ropponen J.O., Jeltsch M., Jokinen J.J., Yla-Herttuala S., Nykanen A.I., Lemstrom K.B. (2012). Critical Role of VEGF-C/VEGFR-3 Signaling in Innate and Adaptive Immune Responses in Experimental Obliterative Bronchiolitis. Am. J. Pathol..

[91] Ristimäki A., Narko K., Enholm B., Joukov V., Alitalo K. (1998). Proinflammatory Cytokines Regulate Expression of the Lymphatic Endothelial Mitogen Vascular Endothelial Growth Factor-C. J. Biol Chem..

[92] Lackner M., Schmotz C., Jeltsch M. (2019). The Proteolytic Activation of Vascular Endothelial Growth Factor-C. LymphForsch.

[93] Huggenberger R., Siddiqui S.S., Brander D., Ullmann S., Zimmermann K., Antsiferova M., Werner S., Alitalo K., Detmar M. (2011). An Important Role of Lymphatic Vessel Activation in Limiting Acute Inflammation. Blood.

[94] Zhou Q., Guo R., Wood R., Boyce B.F., Liang Q., Wang Y.-J., Schwarz E.M., Xing L. (2011). VEGF-C Attenuates Joint Damage in Chronic Inflammatory Arthritis by Accelerating Local Lymphatic Drainage. Arthritis Rheum.

[95] Christiansen A.J., Dieterich L.C., Ohs I., Bachmann S.B., Bianchi R., Proulx S.T., Hollmén M., Aebischer D., Detmar M. (2016). Lymphatic Endothelial Cells Attenuate Inflammation via Suppression of Dendritic Cell Maturation. Oncotarget.

[96] Planas-Paz L., Lammert E. (2013). Mechanical Forces in Lymphatic Vascular Development and Disease. Cell. Mol. Life Sci..

[97] Yamamura A., Nayeem M.J., Muramatsu H., Nakamura K., Sato M. (2021). MAZ51 Blocks the Tumor Growth of Prostate Cancer by Inhibiting Vascular Endothelial Growth Factor Receptor 3. Front. Pharmacol..

[98] Joukov V., Pajusola K., Kaipainen A., Chilov D., Lahtinen I., Kukk E., Saksela O., Kalkkinen N., Alitalo K. (1996). A Novel Vascular Endothelial Growth Factor, VEGF-C, Is a Ligand for the Flt4 (VEGFR-3) and KDR (VEGFR-2) Receptor Tyrosine Kinases. EMBO J..

[99] Woollard D.J., Opeskin K., Coso S., Wu D., Baldwin M.E., Williams E.D. (2013). Differential Expression of VEGF Ligands and Receptors in Prostate Cancer. Prostate.

[100] De Brot S., Ntekim A., Cardenas R., James V., Allegrucci C., Heery D.M., Bates D.O., Ødum N., Persson J.L., Mongan N.P. (2015). Regulation of Vascular Endothelial Growth Factor in Prostate Cancer. Endocr. Relat. Cancer.

[101] Rivera P.J., del Monter V.M.R., Barrientos A.C., Toscano G.J.D., Cuesta M.T., Flores E.J. (2018). Evaluation of VEGF and PEDF in Prostate Cancer: A Preliminary Study in Serum and Biopsies. Oncol. Lett..

[102] Häggström S., Lissbrant I.F., Bergh A., Damber J.E. (1999). Testosterone Induces Vascular Endothelial Growth Factor Synthesis in the Ventral Prostate in Castrated Rats. J. Urol..

[103] Godoy A., Montecinos V.P., Gray D.R., Sotomayor P., Yau J.M., Vethanayagam R.R., Singh S., Mohler J.L., Smith G.J. (2011). Androgen Deprivation Induces Rapid Involution and Recovery of Human Prostate Vasculature. Am. J. Physiol. Endocrinol. Metab..

[104] Wang G., Kovalenko B., Huang Y., Moscatelli D. (2007). Vascular Endothelial Growth Factor and Angiopoietin Are Required for Prostate Regeneration. Prostate.

[105] Morfoisse F., Zamora A., Marchaud E., Nougue M., Diallo L.H., David F., Roussel E., Lacazette E., Prats A.-C., Tatin F. (2021). Sex Hormones in Lymphedema. Cancers.

[106] Asai A., Miyata Y., Matsuo T., Shida Y., Hakariya T., Ohba K., Sakai H. (2017). Changes in Lymphangiogenesis and Vascular Endothelial Growth Factor Expression by Neo-----Adjuvant Hormonal Therapy in Prostate Cancer Patients. Prostate.

[107] Tuomela J., Valta M., Seppänen J., Tarkkonen K., Väänänen H.K., Härkönen P. (2009). Overexpression of Vascular Endothelial Growth Factor C Increases Growth and Alters the Metastatic Pattern of Orthotopic PC-3 Prostate Tumors. BMC Cancer.

[108] Cursiefen C., Chen L., Borges L.P., Jackson D., Cao J., Radziejewski C., D’Amore P.A., Dana M.R., Wiegand S.J., Streilein J.W. (2004). VEGF-A Stimulates Lymphangiogenesis and Hemangiogenesis in Inflammatory Neovascularization via Macrophage Recruitment. J. Clin. Investig..

[109] Schoppmann S.F., Birner P., Stöckl J., Kalt R., Ullrich R., Caucig C., Kriehuber E., Nagy K., Alitalo K., Kerjaschki D. (2002). Tumor-Associated Macrophages Express Lymphatic Endothelial Growth Factors and Are Related to Peritumoral Lymphangiogenesis. Am. J. Pathol..

[110] Yuri P., Shigemura K., Kitagawa K., Hadibrata E., Risan M., Zulfiqqar A., Soeroharjo I., Hendri A.Z., Danarto R., Ishii A. (2020). Increased Tumor-Associated Macrophages in the Prostate Cancer Microenvironment Predicted Patients’ Survival and Responses to Androgen Deprivation Therapies in Indonesian Patients Cohort. Prostate Int..

[111] Morfoisse F., Tatin F., Chaput B., Therville N., Vaysse C., Métivier R., Malloizel-Delaunay J., Pujol F., Godet A.-C., De Toni F. (2018). Lymphatic Vasculature Requires Estrogen Receptor-α Signaling to Protect from Lymphedema. Arterioscler. Thromb. Vasc. Biol..

[112] Kozakowski N., Hartmann C., Klingler H.C., Susani M., Mazal P.R., Scharrer A., Haitel A. (2014). Immunohistochemical Expression of PDGFR, VEGF-C, and Proteins of the MToR Pathway before and after Androgen Deprivation Therapy in Prostate Carcinoma: Significant Decrease after Treatment. Targ. Oncol..

[113] Miyata Y., Nakamura Y., Yasuda T., Matsuo T., Ohba K., Furusato B., Fukuoka J., Sakai H. (2017). Neoadjuvant Hormonal Therapy for Low-Risk Prostate Cancer Induces Biochemical Recurrence after Radical Prostatectomy via Increased Lymphangiogenesis-Related Parameters. Prostate.

[114] Rinaldo F., Li J., Wang E., Muders M., Datta K. (2007). RalA Regulates Vascular Endothelial Growth Factor-C (VEGF-C) Synthesis in Prostate Cancer Cells during Androgen Ablation. Oncogene.

[115] Jennbacken K., Vallbo C., Wang W., Damber J.-E. (2005). Expression of Vascular Endothelial Growth Factor C (VEGF-C) and VEGF Receptor-3 in Human Prostate Cancer Is Associated with Regional Lymph Node Metastasis. Prostate.

[116] Li J., Wang E., Rinaldo F., Datta K. (2005). Upregulation of VEGF-C by Androgen Depletion: The Involvement of IGF-IR-FOXO Pathway. Oncogene.

[117] Yang J., Wu H.-F., Qian L.-X., Zhang W., Hua L.-X., Yu M.-L., Wang Z., Xu Z.-Q., Sui Y.-G., Wang X.-R. (2006). Increased Expressions of Vascular Endothelial Growth Factor (VEGF), VEGF-C and VEGF Receptor-3 in Prostate Cancer Tissue Are Associated with Tumor Progression. Asian J. Androl..

[118] Zhang Z., Luo G., Tang H., Cheng C., Wang P. (2016). Prognostic Significance of High VEGF-C Expression for Patients with Breast Cancer: An Update Meta Analysis. PLoS ONE.

[119] Jha S.K., Rauniyar K., Karpanen T., Leppänen V.-M., Brouillard P., Vikkula M., Alitalo K., Jeltsch M. (2017). Efficient Activation of the Lymphangiogenic Growth Factor VEGF-C Requires the C-Terminal Domain of VEGF-C and the N-Terminal Domain of CCBE1. Sci. Rep..

[120] Stacker S.A., Caesar C., Baldwin M.E., Thornton G.E., Williams R.A., Prevo R., Jackson D.G., Nishikawa S., Kubo H., Achen M.G. (2001). VEGF-D Promotes the Metastatic Spread of Tumor Cells via the Lymphatics. Nat. Med..

[121] Harris N.C., Paavonen K., Davydova N., Roufail S., Sato T., Zhang Y.-F., Karnezis T., Stacker S.A., Achen M.G. (2011). Proteolytic Processing of Vascular Endothelial Growth Factor-D Is Essential for Its Capacity to Promote the Growth and Spread of Cancer. FASEB J..

[122] Carmeliet P., Li X., Treps L., Conradi L.-C., Loges S. (2018). RAISEing VEGF-D’s Importance as Predictive Biomarker for Ramucirumab in Metastatic Colorectal Cancer Patients. Ann. Oncol..

[123] Sharma N.L., Massie C.E., Ramos-Montoya A., Zecchini V., Scott H.E., Lamb A.D., MacArthur S., Stark R., Warren A.Y., Mills I.G. (2013). The androgen receptor induces a distinct transcriptional program in castration-resistant prostate cancer in man. Cancer Cell.

[124] Bhat S.M., Badiger V.A., Vasishta S., Chakraborty J., Prasad S., Ghosh S., Joshi M.B. (2021). 3D Tumor Angiogenesis Models: Recent Advances and Challenges. J. Cancer Res. Clin. Oncol..

[125] Wörsdörfer P., Dalda N., Kern A., Krüger S., Wagner N., Kwok C.K., Henke E., Ergün S. (2019). Generation of Complex Human Organoid Models Including Vascular Networks by Incorporation of Mesodermal Progenitor Cells. Sci. Rep..

[126] Shirure V.S., Hughes C.C.W., George S.C. (2021). Engineering Vascularized Organoid-on-a-Chip Models. Annu. Rev. Biomed. Eng..

[127] Brassard-Jollive N., Monnot C., Muller L., Germain S. (2020). In Vitro 3D Systems to Model Tumor Angiogenesis and Interactions with Stromal Cells. Front. Cell Dev. Biol..

